# Polymorphism *rs1761667* in the *CD36* Gene Is Associated to Changes in Fatty Acid Metabolism and Circulating Endocannabinoid Levels Distinctively in Normal Weight and Obese Subjects

**DOI:** 10.3389/fphys.2017.01006

**Published:** 2017-12-06

**Authors:** Melania Melis, Gianfranca Carta, Stefano Pintus, Paolo Pintus, Carla A. Piras, Elisabetta Murru, Claudia Manca, Vincenzo Di Marzo, Sebastiano Banni, Iole Tomassini Barbarossa

**Affiliations:** ^1^Department of Biomedical Sciences, Section of Physiology, University of Cagliari, Monserrato, Italy; ^2^Department of Internal Medicine, Center for Metabolic Diseases, Azienda Ospedaliera G. Brotzu, Cagliari, Italy; ^3^Endocannabinoid Research Group, Institute of Biomolecular Chemistry, Consiglio Nazionale delle Ricerche, Pozzuoli, Italy

**Keywords:** *CD36* gene, obesity, fatty acids, metabolism, endocannabinoids

## Abstract

The multifunctional CD36 scavenger receptor facilitates fatty acid (FA) uptake and oxidation and it has been involved in the pathophysiology related to dysfunctional FA metabolism. The common variant in the *CD36* gene, *rs1761667* (A/G), whose allele A is characterized by a reduced protein expression, has been associated with taste sensitivity to and preference for fat. We therefore aimed at evaluating whether the *CD36* polymorphism may influence fatty acid metabolism and endocannabinoid biosynthesis in normal weight (NW) and obese (OB) subjects. Red blood cell (RBC) fatty acid composition, and plasma endocannabinoid levels were determined. In NW subjects with AA genotype was found a marked reduction of RBC saturated fatty acids and palmitic/linoleic ratio (PA/LA), considered as *de novo* lipogenesis (DNL) biomarkers. Remarkably, to the reduction of DNL biomarkers corresponded an increase of omega-6 index, an indirect marker of the impact on fatty acid metabolism of dietary omega-6 fatty acids, endocannabinoid levels and a higher waist/hip ratio. The presence of the G allele was instead associated with increased endocannabinoid plasma levels and a trend for increased waist/hip ratio in obese subjects, even though exhibited decreased BMI with respect to those with AA genotype. These data indicate that the *CD36* polymorphism, *rs1761667*, leads to a distinct metabolic pattern in NW and in OB subjects. Therefore, their determination may be crucial in developing personalized therapeutic strategies for ameliorating dyslipidemia and other metabolic disorders.

## Introduction

The multifunctional CD36 scavenger receptor is an 88kDa membrane glycosilated protein of 471 amino acid which has been originally identified in platelet membrane (Ikeda et al., 1989). CD36 is expressed on different cell types, such as adipocytes, myocytes, monocytes, macrophages, hepatocytes, vascular endothelial cells and intestinal enterocytes (Silverstein and Febbraio, 2009; Su and Abumrad, 2009). Topology of this receptor predicts an extracellular domain with one hydrophobic sequence, which may loop back into the membrane, and two shorts cytoplasmic (Su and Abumrad, 2009). In the last decades, several papers have been focused on the role of the scavenger receptor CD36 in facilitating the uptake and oxidation of fatty acid (FA) in humans and rodents and its involvement in the pathophysiological mechanisms associated with dysfunctional FA metabolism (Ma et al., 2004; Love-Gregory et al., 2008; Schwenk et al., 2008; Zhou et al., 2008). In addition to facilitate FA uptake, the membrane protein CD36 acts as a signaling molecule and a receptor for a wide range of ligands (Febbraio and Silverstein, 2007), binds to resident lipoproteins facilitating the cholesteryl ester uptake and enables the uptake of oxidized low/high-density lipoproteins and cholesterol (Calvo et al., 1998; Nassir et al., 2007; Thorne et al., 2007). As a consequence of its many ligands and functions, CD36 could impact on a wide range of dysmetabolic conditions associated with obesity, such as dyslipidaemia, insulin resistance, diabetes, inflammation, atherosclerosis and cancer (Hirano et al., 2003; Pascual et al., 2017).

CD36 plays a decisive role in the orosensory perception of dietary lipids in mammals since long-chain fatty acids appear to be mainly accountable for dietary fat taste perception in the oral cavity (Fukuwatari et al., 2003; Martin et al., 2011; Pepino et al., 2012; Melis et al., 2015; Besnard et al., 2016). CD36 is expressed also on taste bud cells of the circumvallate papillae where it has been demonstrated to mediate the perception of FA and the initiation of the cephalic phase (Laugerette et al., 2005; Sclafani et al., 2007; Martin et al., 2011). CD36 expression in circumvallate papillae has been found to be significantly decreased in high-fat diet-induced obese rats (Zhang et al., 2011). This suggested that decreased expression of CD36 diminishes sensitivity to fat taste, leading to increase fatty food intake as a compensatory reaction. On the other hand, absolute deficiency seems to decrease fat intake, probably as a post-ingestive effect of late absorption (Sclafani et al., 2007).

The presence of CD36 has been found in gustatory papillae in humans (Simons et al., 2011; Ozdener et al., 2014) as the main long-chain fatty acid receptor in taste bud cells, contributing to the orosensory perception of dietary lipid and fat preference (Keller et al., 2012; Pepino et al., 2012). Greater preference for, and increased eating of fatty foods have been documented in obese subjects (Stewart et al., 2010), which may reflect a reduction of oral and gastrointestinal FA sensitivity in obesity (Stewart et al., 2011).

The polymorphisms of *CD36* gene, *rs1761667, rs1527483*, and *rs3840546*, have been associated with taste perception to and preference for fat (*rs1761667, rs1527483*), and obesity (*rs3840546*) (Keller et al., 2012; Pepino et al., 2012; Daoudi et al., 2015; Melis et al., 2015; Karmous et al., 2017). Several reports highlighted the effect of the polymorphism *rs1761667* on CD36 protein expression levels (Ghosh et al., 2011; Love-Gregory et al., 2011), that could justify changes in oro-sensory perception of fats (Ma et al., 2004; Madden et al., 2008; Baranowski et al., 2011).

Recently, we have found that taste sensitivity for the bitter taste of 6-n-propylthiouracil (PROP), regarded as a general oral marker for chemosensory perception, food preference and BMI (Tepper, 2008; Padiglia et al., 2010; Tepper et al., 2011, 2014; Melis and Tomassini Barbarossa, 2017; Melis et al., 2017), affects lipid metabolism in normal weight (NW) (Tomassini Barbarossa et al., 2013) and obese (OB) (Carta et al., 2017) subjects. Peculiarly, in subjects with obesity, we showed an increased BMI and decreased levels of plasmatic endocannabinoids in subjects with extremely elevated PROP sensitivity, also named super-testers, vs. subjects with reduced PROP sensitivity (non-tasters). However, OB super-tasters showed lower circulating non-esterified fatty acid (NEFA) and retinol levels, which may indicate a more favorable lipid metabolism and body fat distribution than those of OB non-tasters. Since circulating levels of endocannabinoids are strongly associated to visceral obesity, less efficient mitochondrial function and ectopic fat deposition (Cristino et al., 2014), these results suggested that PROP taste sensitivity may predispose to metabolic changes and thereby body mass composition. Therefore, due to the relevant influence of the *CD36* polymorphism *rs1761667* on macronutrient preference and cellular lipid metabolism in humans, we aimed at evaluating whether it may impact fatty acid metabolism and endocannabinoid biosynthesis in NW and OB subjects.

## Materials and methods

### Participants

126 Caucasian volunteers (46 M and 80 F) were recruited through public advertisements in the area of Cagliari, Italy. Subjects were divided into two groups based on their BMI: Normal weight subjects (NW) had a BMI ranging from 18 to 25 kg/m^2^ (*N* = 64; 21 M, 43 F) and subjects with obesity (OB) had a BMI ranging from 30 to 50 kg/m^2^ (*N* = 62; 25 M, 37 F).

Participants with major diseases (e.g., diabetes and kidney disease), pregnancy or lactation, food allergies and those who used medications that could affect lipid metabolism (e.g., steroids, antihistamines, and certain antidepressants) were excluded. All subjects were informed concerning the procedure and the purpose of the study. All participants provided a signed informed consent form. The study was performed according to the guidelines of the Declaration of Helsinki of 1975 (revised in 1983), and the procedures involving human participants were approved by the ethical committee of the Brotzu Institution. The present study has been registered at ClinicalTrials.gov (Identifer: NCT02729584).

### Study design

All subjects were requested to refrain from eating and drinking (except water) for at least 8 h prior to the sample withdrawal. They had to be in the test room at 8.00 a.m. In each subject, weight (kg) and height (m) were measured and the body mass index (BMI) (kg/m^2^) calculated, and waist and hip circumferences were determined. In each subject, a sample of blood (4-mL) was collected, promptly centrifuged and stored at −80°C until the analyses were completed as described below. Afterwards, a 2-mL sample of saliva was collected and transferred into an acid-washed polypropylene test tube. The saliva samples were stored at −80°C until molecular analyses were completed, as described below.

### Molecular analyses

DNA was extracted from each saliva sample by using the QIAamp ® DNA Mini Kit (QIAGEN Hilden, Germany) according to the manufacturer's instructions. Its concentration was assessed by measurements at an optical density of 260 nm with an Agilent Cary 60 Uv-Vis Spectrophotometer (Agilent, Palo Alto, CA).

Subjects were genotyped for the single nucleotide polymorphism (SNP), *rs1761667* (G/A), of *CD36*, located at the −31118 promoter region of exon 1A.

A polymerase chain reaction (PCR) was used to amplify the *CD36* region, including the polymorphism. The forward 5′-CAAAATCACAATCTATTCAAGACCA-3′ and reverse 5′-TTTTGGGAGAAATTCTGAAGAG-3′primers were synthesized by Invitrogen (50 nmol scale, desalted) (Europrim, Invitrogen Cambridge, UK). To genotype the *CD36 rs1761667* polymorphism molecular analyses were carried out by PCR followed by analysis with restriction enzyme of the fragments obtained according to Banerjee et al. (2010), as briefly described below. A190-bp fragment was amplified with the primers and digested with the HhaI restriction enzyme (Thermo Scientific Inc, Waltham, USA). This restriction enzyme recognizes GCG^∧^C site and cut if incubated at 37°C overnight. Digestion products were separated by electrophoresis on a 2% agarose gel and the DNA bands were visualized by ethidium bromide staining and ultraviolet light to score the deletion. PCR 50 bp Low Ladder DNA was used as a molecular mass marker (Gene Ruler™ -Thermo Scientific).

### Samples preparation, lipid, and endocannabinoid extraction

Blood samples were taken from the antecubital veins of volunteers, EDTA (10 IU/mL) was added to blood samples and promptly centrifuged at 2,000 g for 10 min, and the resulting plasma and erythrocytes were stored at −80°C until analysis.

Plasma or erythrocytes (red blood cells, RBC) samples were homogenized and extracted with a trichloromethane-methanol solution (2:1, v/v) (Folch et al., 1957) containing 2 μg of vitamin E and internal deuterated standards, anandamide (AEA), 2-arachidonoyl-monoacylglycerol (2-AG), palmitoylethanolamide (PEA) and oleoylethanolamide (OEA), for quantification by isotope dilution ([2H]^8^ AEA, [2H]^5^ 2-AG, [2H]^4^ PEA, and [2H]^4^ OEA as deuterated isotopes; Cayman Chemical, MI, USA; Piscitelli et al., 2011).

#### Analysis of the endocannabinoids and their congeners in plasma

Aliquots from the lipid-containing organic phase, from plasma, were used for AEA, 2-AG, PEA, and OEA quantification by liquid chromatography–atmospheric pressure chemical ionization–mass spectrometry [1100 HPLC system (Agilent Technologies, Santa Clara, CA, USA) equipped with MS Detector 6110 single quadrupole] by selected ion monitoring at M+1 values for the four compounds and their deuterated homologs, as described previously (Piscitelli et al., 2011).

#### Analysis of erythrocytes fatty acids

An aliquot of the lipid fraction, from erythrocyte, was mildly saponified as described by Banni et al. (1996) in order to obtain free fatty acids (FFA) for HPLC analysis. Separation of FFA was performed with Agilent 1100 HPLC system equipped with a diode array detector (Agilent, Palo Alto, CA), a C-18 Inertsil 5 ODS-2 Chrompack column (Chrompack International BV, The Netherlands), 5 μm particle size, 150 4.6 mm, was used with a mobile phase of CH3CN/H2O/CHCOOH (70/30/0.12, v/v/v) at a flow rate of 1.5 mL/min as described in Melis et al. (2001). Unsaturated FFA were detected at 200 nm; spectra (195–315 nm) of the eluate were obtained every 1.28 s and were electronically stored, HP Chemstation software (Agilent, Palo Alto, CA). These spectra were taken to validate the identification of the HPLC peaks (Angioni et al., 2002).

Because saturated fatty acid (SAFA) are transparent to UV, after derivatization, they were analyzed as fatty acid methyl esters by a gas chromatograph with flame ionization detector (FID) (Agilent, Model 6890, Palo Alto, CA) as described previously in Batetta et al. (2009). Different fatty acid metabolic parameters were calculated. The ratio of the concentration of PA/LA and PA/ARA were taken as an indirect measurement of *de novo* lipogenesis (Jacobs et al., 2015). Omega-6 index, taken as an index of the impact of dietary intake of omega-6 on erythrocyte concentrations of omega-6 highly polyunsaturated fatty acids (HPUFAs), was calculated as the ratio between the sum of the concentrations of omega-6 HPUFAs (those with 20 or more carbon atoms and three or more double bonds) and the sum of total fatty acids: (20:4n-6+20:3n-6+22:4n-6+22:5n-6)/(sum of total fatty acids), similarly to how the omega-3 index is calculated (Harris and Von Schacky, 2004).

### Statistical analyses

Genotype distribution and allele frequencies of the *CD36* SNP between NW and OB was tested by the Fisher method. The data are expressed as the mean ± SEM. A two-way analysis of covariance (ANCOVA) was used to analyze the differences for all parameters associated to genotypes of the *CD36* SNP in NW and OB, where age was used as a covariate. Post hoc comparisons were performed with the Fisher least significant difference (LSD) test, unless the assumption of homogeneity of variance was violated, in which case the Duncan's test was used. Statistical analyses were conducted using the software STATISTICA for WINDOWS (version 7; StatSoft Inc, Tulsa, OK, USA). *P* ≤ 0.05 were considered significant.

## Results

Molecular analysis at the *rs1761667* (A/G) polymorphism of *CD36* identified in the group of NW seven subjects being homozygous AA, 39 heterozygous AG, and 18 homozygous GG, while in OB, 15 subjects were homozygous AA, 31 heterozygous AG, and 16 homozygous GG. No differences were found between NW and OB according to the genotype distribution and allele frequency of this polymorphism (χ^2^ < 2.7282; *p* > 0.2308). However, it is interesting to note that the number of subjects who had the AA genotype was more than two-fold in OB (24%) than in NW (10%) (χ^2^ = 3.384; *p* = 0.05).

**Figure 1:**
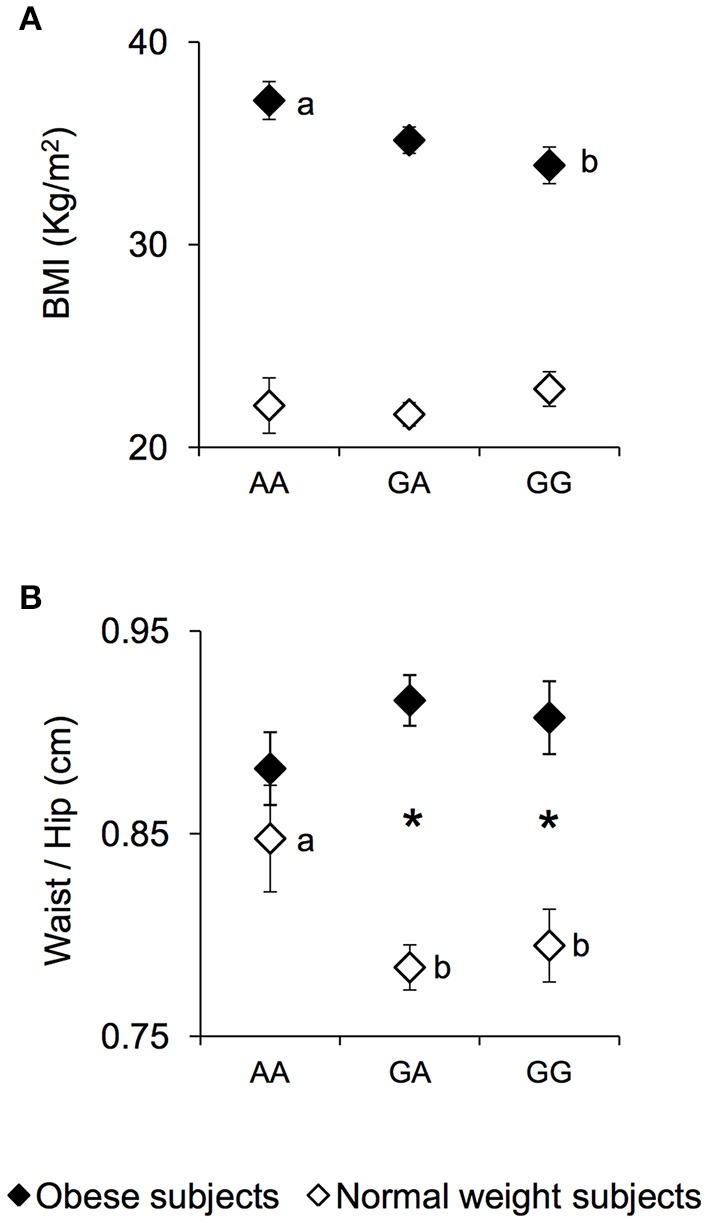
BMI and waist/hip ratio according to the *rs1761667* (A/G) polymorphism of *CD36* in normal weight and obese subjects. Mean values ± SEM of body mass index (BMI) **(A)** and the waist/hip ratio **(B)** of homozygous AA at the polymorphism of *CD36* (*N* = 7), heterozygous (*N* = 39) and homozygous GG (*N* = 18) subjects with normal weight (NW) and homozygous AA (*N* = 15), heterozygous (*N* = 31) and homozygous GG (*N* = 16) subjects with obesity (OB). The different letters indicate significant differences (BMI: *p* < 0.0419; waist/hip ratio: *p* < 0.040; Duncan test subsequent two-way ANCOVA). ^*^a significant difference between corresponding NW or OB subjects (waist/hip ratio: *p* < 0.000085; Duncan test subsequent two-way ANCOVA).

The mean values ± SEM of BMI and the waist/hip ratio, in NW and OB subjects, according to genotypes of *rs1761667* locus are shown in Figure 1. Post hoc comparisons subsequent to two-way ANCOVA showed that OB subjects homozygous for the G-allele exhibited a BMI lower than OB subjects being homozygous AA (*p* = 0.019, Fisher LSD test) (Figure 1A). An opposite trend, although not significant, was found in NW (Figure 1A). A pairwise comparison also showed that the waist/hip ratio of homozygous AA and NW subjects was significantly higher than that of NW subjects that were heterozygous or homozygous for the G-allele (*p* < 0.040, Duncan test), and not significantly different from that determined in OB subjects with the same genotype, who exhibited values not different from those of other genotype groups of OB subjects (Figure 1B). On the other hand, heterozygous or homozygous for the G-allele OB subjects still showed a higher waist/hip ratio than NW subjects with the same genotype (*p* < 0.000085, Duncan test) (Figure 1B).

**Figure 2:**
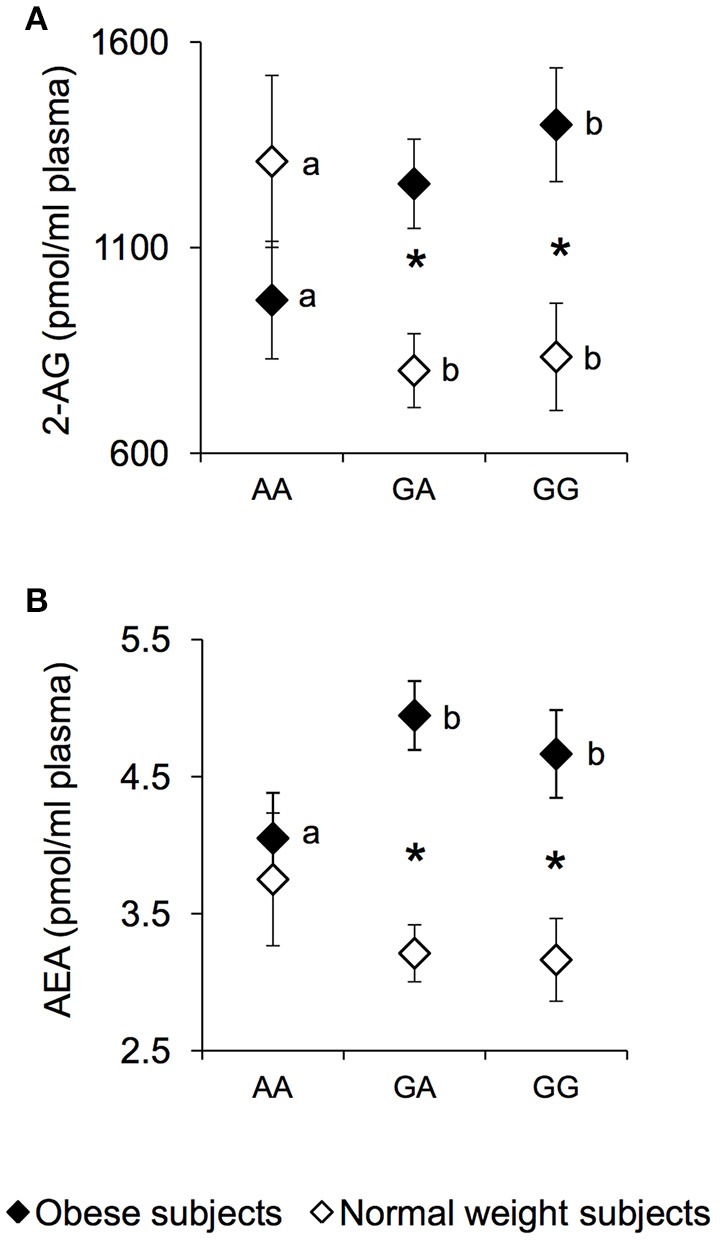
2-AG and AEA according to the *rs1761667* (A/G) polymorphism of *CD36* in normal weight and obese subjects. Mean values ± SEM of endocannabinoids (AEA and 2-AG) plasma levels measured in homozygous AA at the polymorphism of *CD36* (*N* = 7), heterozygous *(N* = 39) and homozygous GG *(N* = 18) subjects with normal weight (NW) and homozygous AA (*N* = 15), heterozygous (*N* = 31) and homozygous GG (*N* = 16) subjects with obesity (OB). **(A)**: 2-arachidonoylglycerol (2-AG) values. **(B)**: anandamide (AEA) values. The different letters indicate significant differences (2-AG: *p* < 0.05; AEA: *p* < 0.05; Fisher LSD test subsequent two-way ANCOVA). ^*^a significant difference between corresponding NW or OB subjects (2-AG: *p* < 0.0016; AEA: *p* < 0.00089; Fisher LSD test subsequent two-way ANCOVA).

The plasma levels of the endocannabinoids 2-AG and AEA were measured in NW and OB according to genotypes of *rs1761667* locus and are presented in Figure 2. An ANCOVA revealed a significant two-way interaction of genotype group × NW/OB status for the 2-AG value [*F*_(2, 119)_ = 4.1645; *p* = 0.0179] (Figure 2A). Post hoc comparisons revealed that 2-AG plasma levels of homozygous AA and NW subjects were significantly higher than those determined in NW subjects that were heterozygous or homozygous for the G-allele (*p* < 0.050, Fisher LSD test), and not significantly different from those of AA and OB subjects, who had values lower than those of homozygous GG OB subjects (*p* = 0.034, Fisher LSD test; Figure 2A). In addition, OB subjects heterozygous or homozygous for the G-allele showed higher 2-AG values than corresponding NW subjects (*p* < 0.000085, Fisher LSD test; Figure 2A). Post hoc comparisons also showed that the AEA plasma levels in homozygous AA and OB subjects were significantly lower than those determined in OB subjects who were heterozygous or homozygous for the G-allele (*p* < 0.050, Fisher LSD test; Figure 2B). An opposite trend, although not significant, was found in NW subjects (Figure 2B). OB subjects heterozygous or homozygous for the G-allele, showed higher AEA values than corresponding NW subjects (*p* < 0.00089, Fisher LSD test; Figure 2B), while the values of homozygous AA OB did not vary with respect to those of homozygous AA NW subjects (Figure 2B). No significant changes of endocannabinoid congeners OEA and PEA were found between NW and OB and *rs1761667* genotype (data not shown).

**Figure 3:**
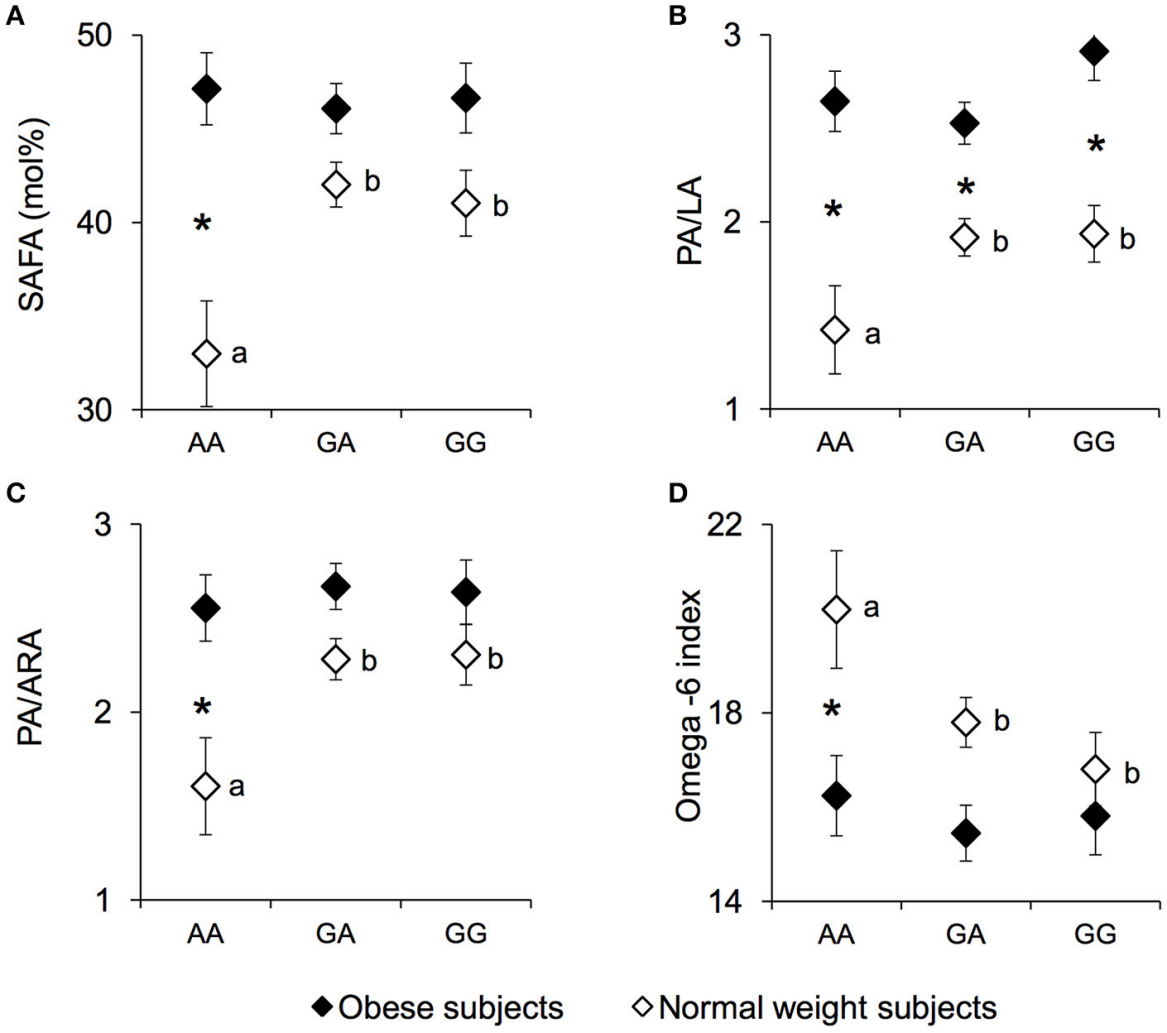
SAFA, PA/LA, PA/ARA, and omega-6 index according to the *rs1761667* (A/G) polymorphism of *CD36* in normal weight and obese subjects. Mean values ± SEM of levels of erythrocyte saturated fatty acid (SAFA), the palmitic/linoleic acids ratio (PA/LA), the palmitic/arachidonic acids ratio (PA/ARA), omega-6 index [(20:4n-6+20:3n-6+22:4n-6+22:5n-6)/(sum of total fatty acids)] measured in homozygous AA at the polymorphism of *CD36* (*N* = 7), heterozygous (*N* = 39) and homozygous GG (*N* = 18) subjects with normal weight (NW) and homozygous AA (*N* = 15), heterozygous (*N* = 31) and homozygous GG (*N* = 16) subjects with obesity (OB). **(A)**: erythrocyte SAFA values. **(B)**: erythrocyte palmitic/linoleic acids ratio (PA/LA). **(C)**: erythrocyte palmitic/arachidonic acids ratio (PA/ARA). **(D)**: omega-6 index values. The different letters indicate significant differences (SAFA: *p* < 0.033; PA/LA: *p* < 0.031; PA/ARA: *p* < 0.0070; omega-6 index: *p* = 0.045 Duncan test). ^*^a significant difference between corresponding NW or OB subjects (SAFA: *p* < 0.000026; PA/LA: *p* < 0.010; PA/ARA: *p* < 0.00037; omega-6 index: *p* = 0.0020 Duncan test).

The erythrocyte SAFA levels (mean values ± SEM), PA/LA ratio, palmitic/arachidonic acid (PA/ARA) ratio, and the omega-6 index measured in NW and OB according to genotypes of the *rs1761667* locus are shown in Figure 3. A significant two-way interaction of genotype group × NW/OB status on SAFA values was found [*F*_(2, 119)_ = 3.7075, *p* = 0.02752; two-way ANCOVA]. Post hoc comparisons showed that the SAFA values (Figure 3A), the PA/LA ratio (Figure 3B) and the PA/ARA ratio (Figure 3C) of NW homozygous for the A-allele were significantly lower than those of NW subjects that were heterozygous or homozygous GG (*p* < 0.029 and *p* < 0.031, respectively; Duncan test). Accordingly, the omega-6 index (Figure 3D) of homozygous for the A-allele NW subjects was significantly higher than that of heterozygous or homozygous GG NW subjects (*p* = 0.045 and *p* = 0.0069, respectively; Duncan test). No significant differences in SAFA values, PA/LA ratios, PA/ARA ratios or the omega-6 index related to *rs1761667* locus were found in OB subjects (*p* > 0.05). The SAFA values, the PA/LA ratio and the PA/ARA ratio of homozygous for the A-allele NW subjects were significantly lower, while the omega-6 index was significantly higher, than those of the corresponding OB subjects (SAFA values: *p* = 0.000026; PA/LA: *p* = 0.000029; PA/ARA: *p* = 0.00037; Omega-6 index: *p* = 0.0020; Duncan test). The PA/LA ratio of OB subjects that were heterozygous or homozygous GG OB was higher than that of the corresponding NW subjects *(p* < 0.010; Duncan test). Concentrations of other fatty acids, as well related metabolic parameters such as omega-3 index, were not found significantly different between NW and OB and *rs1761667* genotype (data not shown).

## Discussion

Our results confirm our previous reports (Tomassini Barbarossa et al., 2013; Carta et al., 2017) that genetic factors influencing dietary preferences affect fatty acid metabolism and circulating levels of endocannabinoids, differently in NW and OB subjects. Interestingly, in NW with the AA genotype of the *rs1761667* polymorphism in *CD36*, characterized by a lower expression of CD36 protein with respect to subjects carrying the G allele, as previously shown (Ghosh et al., 2011; Love-Gregory et al., 2011), we found a marked reduction of RBC SAFA levels and PA/LA and PA/ARA ratios which have been used as markers of DNL (Jacobs et al., 2015). DNL is a metabolic pathway characterized by PA formation from glucose (Vernieri et al., 2016). Thereby biomarkers of DNL has also been taken as markers of carbohydrate intake (Jacobs et al., 2015). In addition, detection of these biomarkers in fatty acid profile of RBC has the advantage to represent long term metabolic changes because it is more steady than that of the plasma (Harris and Thomas, 2010). Therefore, we may hypothesize that NW individuals with AA genotype have a lower intake of carbohydrates compared to individuals NW carrying the G allele in the same locus.

Remarkably, to the reduction of DNL biomarkers corresponded an increase of the omega-6 index, which has been proposed as a marker of fat intake (Hudgins et al., 1996; Raatz et al., 2001; Chong et al., 2008; Jacobs et al., 2015). Indeed, individuals with the AA genotype show a preference for dietary fat (Keller et al., 2012). Thus, higher 2-AG levels in NW subjects with AA genotype may indicate that 2-AG biosynthesis is influenced by a high fat intake, particularly omega-6 fatty acids. In fact, 2-AG is ultimately derived from the omega-6 polyunsaturated fatty acid arachidonic acid and its biosynthetic precursor LA, and it has been shown in different experimental models (Matias et al., 2008; Batetta et al., 2009; Di Marzo et al., 2010; Piscitelli et al., 2011; Alvheim et al., 2012; Demizieux et al., 2016) and in humans (Banni et al., 2011), that changes in the precursor availability significantly affect endocannabinoids tissue levels. AEA circulating levels showed a similar trend, which did not reach statistical significance suggesting that other variables may influence the circulating levels of this endocannabinoid. Importantly, NW subjects with the AA genotype were also characterized by a higher waist/hip ratio, in agreement with data in the literature showing that in humans and experimental models, elevated endocannabinoid levels are associated to visceral fat deposition (Côté et al., 2007; Di Marzo et al., 2009). In obese subjects, however, AA genotype, was not associated to changes in fatty acid profile biomarkers of intake of carbohydrates or fat, in particular the omega-6 index. A possible explanation could be a deranged glucose disposal in obese subjects, irrespective of the genotype, which may enhance DNL, as revealed by the specific biomarker PA/LA. On the other hand, it is less intuitive that in obese subjects carrying the G allele again exhibit increased endocannabinoids plasma levels, with respect to those of OB subjects with the AA genotype. *CD36* GG genotype is characterized by a highly expressed CD36 receptor which has a significant role in the regulation of fatty acid entry into the cell in physiological conditions. However, it has been shown that in conditions of an excess fatty acid entry, as it occurs in obese subjects with higher circulating levels of NEFA, CD36 activity is impaired, inducing ectopic fat deposition and decreasing mitochondrial activity (Pepino et al., 2014; Samovski et al., 2015) leading to a reduction of lean mass and increasing fat mass particularly in the visceral adipose tissue. This hypothesis is supported by the decrease of BMI, possibly due to a reduction in lean mass, which corresponded to a trend for increased waist/hip ratio in GG and GA OB subjects when compared to those carrying the AA genotype, which is also more frequent in OB subject than in NW. Thus, while in NW subjects with the AA genotype of *CD36* the increased 2-AG plasma levels might be due at least in part to increased omega-6 intake and reduced DNL, in OB subjects with the GG or GA phenotype the excess visceral fat might become a source of endocannabinoids.

We may therefore conclude that a reduced function due to a low expression of CD 36 protein, either for genetic reason, as in the AA genotype, or due to an excess exposure to fatty acids as it occurs in metabolically dysfunctional OB subjects, may lead to impaired fat disposal at the cellular and tissue level and increased endocannabinoid signaling at cannabinoid CB1 receptors. The latter effect may predispose NW subjects carrying the AA allele of *CD36* to become obese, and set into motion a feedforward vicious circle leading to more visceral fat accumulation in OB subjects carrying the GG allele. At any rate, our data indicate that in the obese state, dysmetabolic conditions influence CD36 activity differently according to its genotype. It has been shown that CD36 activity may also influence the metastatic properties of tumor cells (Pascual et al., 2017), implicated in the incorporation of oxidized LDL in macrophages during atherosclerotic plaque formation (Park et al., 2009) and has been associated to Alzheimer disease (Šerý et al., 2017). Conversely, in physiological conditions a functional CD36 regulates, in a concerted action with AMPK, fatty acid entry and mitochondrial beta-oxidation in the muscle and heart (Pepino et al., 2014; Samovski et al., 2015), even though excessive fatty acid exposure impairs AMPK activity (Samovski et al., 2015) and may thereby lead to metabolic derangement. This implies that CD36 plays a key role in cellular fatty acid metabolism in physiological and pathological conditions in different tissues. Indeed, *CD36* knockout mice are resistant to obesity and dysmetabolic disorders (Clugston et al., 2014).

In conclusion, the A/G allele of the *rs1761667* polymorphism of *CD36* was found associated to a distinct metabolic pattern in NW and obese subjects. Therefore, the identification of the latter may be crucial in developing personalized therapeutic strategies for ameliorating dyslipidemia and other metabolic disorders.

## Author contributions

Designed the work: SB, IT; data acquisition: GC, MM, CM, EM, CP, SP, and PP; analysis and interpretation of data: MM, IT, SB, and GC; drafting the work: SB, IT; revising critically the work: SB, IT, MM, EM, CP, GC, and VD; final approval of the version to be published: SB, IT, MM, GC, CM, EM, CP, and VD; agree to be accountable for all aspects of the work in ensuring that questions related to the accuracy or integrity of any part of the work were appropriately investigated and resolved: SB, IT, MM, GC, CM, EM, CP, and VD.

### Conflict of interest statement

The authors declare that the research was conducted in the absence of any commercial or financial relationships that could be construed as a potential conflict of interest.

## Abbreviations

2-AG: 2-arachidonoyl-monoacylglycerol
AEA: Anandamide
ARA: arachidonic acid
DNL: de novo lipogenesis
FA: fatty acid
FFA: free fatty acid
LA: linoleic acid
NEFA: non esterified fatty acid
NW: normal weight subjects
OB: obese subjects
OEA: oleoylethanolamide
PA: palmitic acid
PEA: palmitoylethanolamide
PROP: 6-n-propylthiouracil
RBC: red blood cell
SAFA: saturated fatty acid.
